# Family Integrated Care (FICare) in Level II Neonatal Intensive Care Units: study protocol for a cluster randomized controlled trial

**DOI:** 10.1186/s13063-017-2181-3

**Published:** 2017-10-10

**Authors:** Karen M. Benzies, Vibhuti Shah, Khalid Aziz, Wanrudee Isaranuwatchai, Luz Palacio-Derflingher, Jeanne Scotland, Jill Larocque, Kelly Mrklas, Esther Suter, Christopher Naugler, Henry T. Stelfox, Radha Chari, Abhay Lodha, Alberta FICare Level II NICU Study Team

**Affiliations:** 1 Faculty of Nursing, Department of Paediatrics, University of Calgary, PF 2278, 2500 University Drive, NW, Calgary, AB T2N 1N4 Canada; 2 Mount Sinai Hospital, Department of Paediatrics and Institute of Health Policy, Management and Evaluation, University of Toronto, Rm 19–231N, Mount Sinai Hospital, 600 University Avenue, Toronto, ON M5G 1X5 Canada; 3 Edmonton Neonatal Program, Department of Pediatrics, Faculty of Medicine and Dentistry, University of Alberta, DTC 5027, Royal Alexandra Hospital, 10240 Kingsway NW, Edmonton, AB T5H 3V9 Canada; 4 Centre for Excellence in Economic Analysis Research (CLEAR), Li Ka Shing Knowledge Institute, St. Michael’s Hospital, Institute of Health Policy, Management and Evaluation, University of Toronto, 30 Bond Street, Toronto, ON M5B 1W8 Canada; 5 Faculty of Kinesiology, Sport Injury Prevention Research Center, Department of Community Health Sciences, University of Calgary, 2500 University Drive NW, PF2250G, Calgary, AB T2N 1N4 Canada; 6 Rockyview General Hospital, Unit 63, 7007 14th Street SW, Calgary, AB T2V 1P9 Canada; 7 DTC 5027, Royal Alexandra Hospital, 10240 Kingsway NW, Edmonton, AB T5H 3V9 Canada; 8 Knowledge for Change Unit, Research Innovation and Analytics, Alberta Health Services, 1103 South Tower, Foothills Medical Centre, 1403 29th Street NW, Calgary, AB T2N 2T9 Canada; 9 Faculty of Social Work, University of Calgary, Calgary, AB T2N 1N4 Canada; 10 Pathology and Laboratory Medicine, Faculty of Medicine, University of Calgary, Calgary, AB T2L 2K8 Canada; 11 General Pathology, Calgary Zone, Alberta Health Services; Calgary Laboratory Services, Alberta Health Services Laboratory Utilization Office, 9-3535 Research Road NW, Calgary, AB T2L 2K8 Canada; 12 Critical Care Strategic Clinical Network, Departments of Critical Care Medicine, Medicine and Community Health Sciences, University of Calgary, 3E18D, TRW Building, 3280 Hospital Drive NW, Calgary, AB T2N 4Z6 Canada; 13 Department of Obstetrics and Gynecology, Edmonton Zone, Alberta Health Services, University of Alberta, 5S131 -10240 Kingsway NW, Edmonton, AB T5H 3V9 Canada; 14 Alberta Health Services, Department of Pediatrics and Community Health Sciences, University of Calgary, C211-1403 29th Street NW, Calgary, AB T2N 2T9 Canada

**Keywords:** Infant, Premature, Family integrated care, Patient engagement, Randomized controlled trial, Parenting education, Nursing, Cost-effectiveness

## Abstract

**Background:**

Every year, about 15 million of the world’s infants are born preterm (before 37 weeks gestation). In Alberta, the preterm birth rate was 8.7% in 2015, the second highest among Canadian provinces. Approximately 20% of preterm infants are born before 32 weeks gestation (early preterm), and require care in a Level III neonatal intensive care unit (NICU); 80% are born moderate (32 weeks and zero days [32^0/7^] to 33^6/7^ weeks) and late preterm (34^0/7^ to 36^6/7^ weeks), and require care in a Level II NICU. Preterm birth and experiences in the NICU disrupt early parent-infant relationships and induce parental psychosocial distress. Family Integrated Care (FICare) shows promise as a model of care in Level III NICUs. The purpose of this study is to evaluate length of stay, infant and maternal clinical outcomes, and costs following adaptation and implementation of FICare in Level II NICUs.

**Methods:**

We will conduct a pragmatic, cluster randomized controlled trial (cRCT) in ten Alberta Level II NICUs allocated to one of two groups: FICare or standard care. The FICare Alberta model involves three theoretically-based, standardized components: information sharing, parenting education, and family support. Our sample size of 181 mother-infant dyads per group is based on the primary outcome of NICU length of stay, 80% participation, and 80% retention at follow-up. Secondary outcomes (e.g., infant clinical outcomes and maternal psychosocial distress) will be assessed shortly after admission to NICU, at discharge and 2 months corrected age. We will conduct economic analysis from two perspectives: the public healthcare payer and society. To understand the utility, acceptability, and impact of FICare, qualitative interviews will be conducted with a subset of mothers at the 2-month follow-up, and with hospital administrators and healthcare providers near the end of the study.

**Discussion:**

Results of this pragmatic cRCT of FICare in Alberta Level II NICUs will inform policy decisions by providing evidence about the clinical effectiveness and costs of FICare.

**Trial registration:**

ClinicalTrials.gov, ID: NCT02879799. Registered on 27 May 2016. Protocol version: 9 June 2016; version 2.

**Electronic supplementary material:**

The online version of this article (doi:10.1186/s13063-017-2181-3) contains supplementary material, which is available to authorized users.

## Background

### Preterm Birth

Each year, about 15 million of the world’s infants are born preterm before 37 weeks gestation [1]. The preterm birth rate in Alberta, 8.7% in 2015, is the second highest among Canadian provinces [2]. These rates may be attributed, in part, to delayed childbearing [3, 4] and assisted reproductive technology [5, 6]. Approximately 20% of preterm infants are born before 32 weeks gestation (early preterm) [7], and require care in a Level III neonatal intensive care unit (NICU) [8]. The remaining 80% are born moderately preterm (32 weeks and zero days [32^0/7^] to 33^6/7^ weeks gestational age [GA]) and late preterm (34^0/7^ to 36^6/7^ weeks GA) [9]. In Alberta, 6.6% of live births, or approximately 3500 infants annually [10], require care in a Level II NICU [8]. Compared with their full-term counterparts, preterm infants who survive are at higher risk for short-term morbidities including respiratory problems [11], hyperbilirubinemia [12], infections [13], and hypoglycemia [12], as well as long-term adverse neurodevelopmental outcomes including cognitive delays [14–16], visual [17, 18] and hearing impairments [18], and behavioral problems [14, 19–21]. Further, as GA decreases, the risk of chronic health problems and developmental delays increases [22, 23].

### Cost of preterm birth

The costs associated with preterm infants are greater than for term infants because of increased (1) hospital length of stay (LOS), (2) resource utilization, (3) readmissions, and (4) need for additional health, education, and social services in the community [24, 25]. In 2010, care of preterm infants represented the largest category of Alberta’s expenditures for pediatric health care at 8.45%, with a total cost of approximately $35 million Canadian Dollar (CAD) [26]. Similar to health consequences of preterm birth, there is a reverse economic gradient associated with GA [27]. That is, the lower the GA, the greater healthcare system and out-of-pocket expenses for parents of preterm infants. In Canada, recently estimated *costs per infant* to age ten years were $67,467 CAD for children born before 28 weeks GA, $52,796 CAD for those born between 28 and 32 weeks GA, and $10,010 CAD for those born between 33 and 36 weeks GA [28]. Financial consequences for parents include reduced workforce participation and lost earnings, as well as the cost of developmental supports for the infant [29]. Unquantifiable costs are associated with psychological distress, marital distress, and social isolation [29]. Although effective psycho-educational interventions for mothers of preterm infants exist [30], evidence is generally limited to the extremely preterm infants and cost-effectiveness data are lacking.

### Level II neonatal intensive care for preterm infants and their families

Level II NICUs are technological, critical care environments where healthcare providers (HCP) often unintentionally marginalize parents in the pursuit of optimal clinical care of preterm infants [31]. The unexpected birth of a preterm infant may leave parents feeling anxious, depressed, isolated, and unprepared to interact with, and care for, their infant [32]. Preterm birth and experiences in the NICU may disrupt breastfeeding [33] and the early parent-infant relationships [34] that are critical for early brain and biological development [35, 36]. Systematic reviews and meta-analyses of interventions for parents of preterm infants suggest that psycho-educational approaches increase maternal self-efficacy, reduce anxiety and depression [37], and increase human-milk feeding [37], with consequent improvements in child development [37]. However, many of these interventions predate evidence of the importance of early parent-child relationships to life-long health [35, 36].

A new model of care, Family Integrated Care (FICare), shows promise as an intervention to improve outcomes for early preterm infants cared for in Level III NICUs [38–40]. Based on these promising results, FICare is being evaluated in two cluster randomized controlled trials (cRCTs): one is a 26-site international (Canada, Australia, New Zealand) trial [41], and one a 6-site trial in China [42]. Another Canadian study using a pre-test/post-test design in Level II NICUs is currently in progress. None of these studies focus on health system impact, nor include an economic analysis. Thus, a pragmatic cRCT [43] is critical to inform health service decisions about clinical effectiveness and costs of FICare for moderate and late preterm infants admitted to Level II NICUs and their families.

### Objectives and hypotheses

The objective of this study is to adapt FICare for implementation in Level II NICUs across Alberta, and evaluate its clinical effectiveness and costs. We hypothesize that FICare will empower parents with the knowledge, skill and confidence to care for their infant(s), and prepare the family for earlier discharge with a concomitant decrease in NICU LOS (primary outcome). Secondarily, we hypothesize that FICare will (1) reduce infant morbidities during hospitalization and the risk of developmental delay at 2 months corrected age (CA), (2) increase rates of human-milk feeding by discharge and at 2 months CA, and (3) reduce parental psychological distress by discharge and at 2 months CA. To understand the utility, acceptability, and impact of FICare, qualitative interviews will be conducted with a subset of mothers at the 2-month-old follow-up, and with hospital administrators and HCP near the end of the study. The specific objectives of the economic analysis are three-fold: (1) to estimate the implementation costs of FICare, (2) to compare public healthcare payer costs of FICare against standard care, and (3) to compare societal costs during hospitalization for parents of FICare against standard care.

## Methods

### Study design

The proposed study employs a pragmatic, parallel-group, superiority cRCT [44] with convenience sampling. A cRCT will avoid contamination between groups, particularly in smaller cities that have only one Level II NICU. The study design and adaptation of the FICare intervention for Alberta Level II NICUs were co-developed by a provincial, multidisciplinary stakeholder group. We adhered to Consolidated Standards of Reporting Trials (CONSORT) and Standard Protocol Items: Recommendations for Interventional Trials (SPIRIT) guidelines in the design of the protocol (Additional file 1). A qualitative component will describe maternal, hospital administrator, and HCP experiences of FICare, the process of implementation itself, and implementation quality.

### Setting

We will conduct the study in ten Level II NICUs (five intervention and five control sites) across the province of Alberta, which is located in Western Canada. Alberta’s population of four million is typically younger with higher income, and a greater proportion of women in their childbearing years than the Canadian average [45]. Alberta has a single, publicly funded health services delivery system (Alberta Health Services [AHS]), which offers advantages for multicenter studies in terms of standardization of many structures and processes across hospitals in the province. All hospitals serve a demographically diverse population that is generally representative of the care of childbearing women in Alberta. AHS HCP must be registered members under the provincial Health Disciplines Act (see http://www.qp.alberta.ca/documents/Acts/H02.pdf). For a list of study sites, see http://www.ucalgary.ca/ficare/ficare-alberta/partnerships.

We will coordinate the trial through a central office in the Faculty of Nursing, University of Calgary. Study coordinators will liaise daily or weekly, as needed, with study nurses to ensure that recruitment targets are being met, data collection is accurate and timely, and fidelity to their group allocation is maintained.

All study nurses (registered or licensed practical nurses) will be employees of AHS, and will receive 4 h of specialized training about (1) the purpose of the FICare study, (2) consenting participants, and (3) data collection. We will provide training “booster doses” when fidelity checks warrant them. For the intervention sites only, we will use a “train-the-trainer” model to provide education about FICare to all HCP at the site. To facilitate study nurses to deliver training at the FICare intervention sites, we will provide an additional 8 h of training about the Alberta Level II NICU FICare model. All HCP in Level II NICUs in this study are generally aware of the principles of patient and family centered care prior to implementation because that is a pillar of care for AHS. However, HCP do not receive specialized training in either family centered care or FICare.

### Eligibility criteria for clusters

All ten Level II NICUs (clusters) across six cities in Alberta were eligible and agreed to participate in the trial. The loss of a cluster would jeopardize completion of this study. To decrease potential loss of sites, we will ensure frequent and effective communication between the coordinating and local sites. To prevent loss of clusters due to: (1) lack of resources, the study will provide a proportion of the salary for a FICare study nurse to each site to support study activities, and (2) resentful demoralization, we budgeted to provide training to control clusters at the end of the study if FICare is effective.

### Eligibility criteria for individual participants

We will include mothers and their preterm infants born between 32^0/7^ weeks to 34^6/7^ weeks GA with a primary admission, or transfer within 72 h, to one of the Level II NICUs. Thirty-two weeks and zero days is the minimum GA for an infant admitted to a Level II NICU; otherwise the infant should be admitted or transferred to a Level III NICU [8]. If otherwise healthy, preterm infants are typically discharged around 36^0/7^ weeks. Thus, 34^6/7^ weeks is the maximum GA at enrollment that will ensure a minimum of 1 week of exposure to FICare. We will confirm GA by first trimester ultrasound or date of last menstrual period, as recorded on the infant’s medical record. We will exclude mothers (1) whose health, social, or language issues may inhibit their ability to communicate with the healthcare team, (2) with triplets or higher-order multiple births who may be overwhelmed with their care, or (3) whose infants require palliative care or have severe congenital or chromosomal anomalies. We will include mothers in the intervention group if they agree to spend a minimum of 6 h per day with their infant.

### Trial arms

#### FICare intervention group

Mothers and their infant(s) in each cluster allocated to the intervention group will receive FICare for Alberta Level II NICUs as the routine care. We adapted the Alberta Level II NICU model of FICare from a four-pillar model established for Level III NICUs [38]. The four-pillar model consists of: (1) staff education and support, (2) parent education, (3) NICU environment, and (4) psychosocial support [38]. Given that (1) preterm infants admitted to a Level II NICU are more mature than infants admitted to a Level III NICU and have a shorter LOS, and (2) because provincial health services are delivered through two providers (Alberta Health Services and Covenant Health), there were questions about the goodness of fit. Following consultation with provincial stakeholders, we reconceptualized the FICare four-pillar model to better fit the Level II NICU population and context for the current cRCT.

FICare for Alberta Level II NICUs is a dynamic, psycho-educational intervention with three main components: (1) information sharing (during one-to-one relational communications and bedside rounds), (2) parent education (one-to-one and group sessions) supported by technology and defined learning pathways, and (3) parental support from professionals (formal) and veteran parents (informal). Veteran parents [46] are those who have had previous experience with their own moderate or late preterm infant in a Level II NICU. The goal of FICare is a change in culture and practice that permits, encourages, and supports parents in their parenting role while their infant is receiving care in a Level II NICU. Underpinned by developmental neuroscience [47], relational communications [48], and adult learning [49] and change [50] theories, FICare empowers parents to sequentially build their knowledge, skill, and confidence so that they are well-prepared to care for their infant long before discharge. This model is dynamic whereby parents and HCP use relational communication skills to negotiate mutually equitable roles during the infant’s NICU stay [48]. Thus, roles change dynamically with the infants’ progress and as parents learn to provide care. For example, at admission nurses may provide 90% of the infant’s care and parents provide 10%; nearer to the time of discharge, parents provide 90% of the care and nurses provides 10%.

#### Standard care control group

Mothers and their infant(s) in the control group will continue to receive standard care. Because this is a pragmatic trial conducted in a progressive and dynamic health system, we will carefully track any changes to policy and practice that may influence outcomes of the FICare study. For example, a secondary outcome of this study is related to frequency of administering incorrect human donor-milk. To reduce donor-milk errors, AHS recently implemented a bar-coding system in several NICUs, including two control and one intervention clusters. We recorded the dates of training and implementation for bar coding and will consider the new procedure when interpreting the results.

#### Contamination of intervention

There are three Level II NICUs in each of the two larger cities, and at least one of the hospitals in each city was randomized to the FICare intervention group. Thus, there is a risk of contamination in the larger cities because casual and part-time nurses and physicians may work at more than one site. We will include nurses in FICare training based on the primary site of their employment. We will ask administrators and HCP in the FICare group not to discuss the study outcomes with colleagues in the standard care group. We will tell families of infants the purpose of the study (to compare different models of care) and requirements to participate, but not give details about FICare. We will carefully track other research studies and practice changes at all sites during the study period to monitor for synergistic and spin-off effects of FICare.

### Site randomization

The biostatistician (LP-D) stratified the ten participating Level II NICUs by size (large and small). Given the desire to have the randomization stratified to ensure balance of standard of care (control) or intervention (FICare) in each stratum, we conducted a simple random sampling within stratum. Given the small sample size in each stratum (four small and six large centers), there was no possibility of creating random-sized blocks to try to do a better allocation concealment or to be able to reduce predictability. Each study group has an equal number of small and large clusters. Group allocation was unknown to any other team member prior to an announcement to provincial stakeholders by LP-D during a telehealth meeting.

#### Blinding

The FICare intervention cannot be blinded; therefore, group allocation cannot be concealed. We will blind data analysts, and the research assistants from the AHS Workforce and Evaluation who are conducting qualitative interviews. We will supervise data analysts, but will not provide any information about the purpose of the study nor group allocation. In the datasets, we will code hospital name and study group to prevent identification.

### Sample size estimate

The sample size estimate for this trial is based on the primary outcome, which is the comparison of LOS between groups. On average the Level II NICU LOS is 16 days; we expect a reduction of 10% (1.6 days) with FICare. Given that LOS has a skewed distribution, we used a natural logarithm transformation to estimate sample size [51]. We estimated sample size based on an inter-class correlation (ICC) of 0.18 (obtained from 2013 aggregated administrative data of the ten participating hospitals), standard deviation (*SD*) in a natural logarithm scale of 0.235 (assuming equal variances between treatment and control groups), a two-tail test with a significance level of 0.05, and a power of 0.80, taking into account unequal cluster sizes [44]. To achieve a power of 0.80 to analyze the primary outcome, we need to approach 227 mothers per group to achieve an effective sample size of 181 per group (total 362).

There are sufficient moderate and late preterm infants to achieve our estimated sample size within 1 year. On average, each Level II NICU unit has 103 infants in our gestation range admitted in 9 months, with a potential total of 1030 infants admitted to the ten sites. With an expected 80% participation rate [38] we will have a potential of 824 mother participants. We will exceed the minimum sample size required to analyze the primary outcome (LOS) so that we can account for those mothers who we recruit and whose infant stays less than 1 week (6%), and to allow us to carry out mixed-effect analysis to answer the primary research question. For the follow-up at 2 months, we estimate an 80% retention rate to follow-up (824 × 0.80 = 659). Thus, our sample size will also allow us to analyze secondary outcomes, including those that will be measured at 2 months.

### Recruitment

We will have similar recruitment procedures across clusters. A study nurse will use a standardized script to inform mothers of the study within 72 h of the birth and primary admission or transfer of their infant(s) to a Level II NICU. For interested mothers, the study nurse will (1) screen for eligibility, (2) answer questions about the study, and (3) obtain written informed consent. Infants of mothers who do not wish to participate in the study will still receive either FICare or standard care, depending on the group allocation of their hospital. We will offer mothers in the intervention group a hospital parking pass to enable them to spend a minimum of 6 h per day in the Level II NICU with their infant(s). We will account for the cost of hospital parking passes for mothers in the intervention group in the economic analyses.

### Participant timeline

**Fig. 1:**
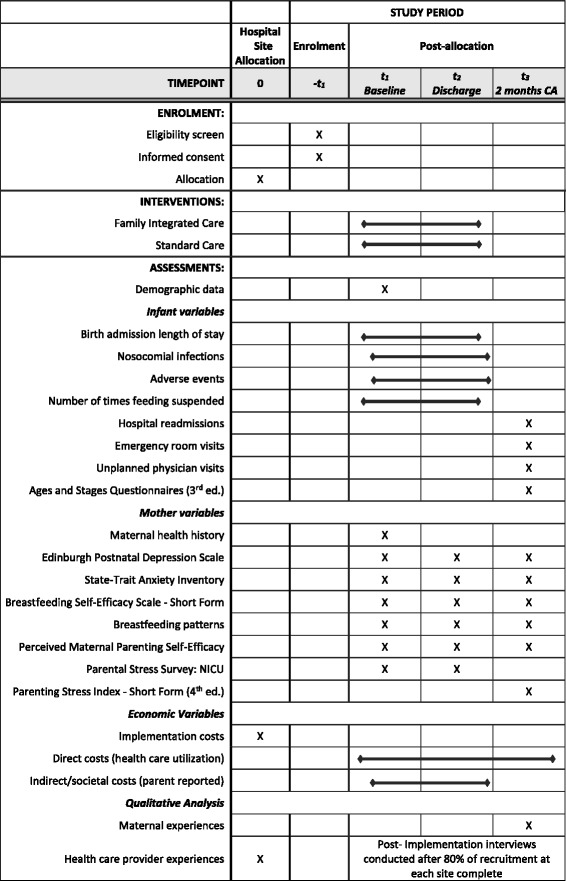
Standard Protocol Items: Recommendations for Interventional Trials (SPIRIT) schedule of enrollment, interventions, and assessments

Mothers in both groups will complete baseline questionnaires as soon as they are well enough, within 72 h of the admission or transfer of their infant(s) to the NICU. The study protocol is based on the SPIRIT Figure (see Fig. 1 for details). Approximately 24 h prior to the expected hospital discharge of their infant(s), mothers will complete outcome questionnaires. Daily, all mothers will complete an investigator-designed (KB and IW) Parent Journal to capture out-of-pocket costs for economic analyses. In addition to cost information, the version of the Parent Journal for the intervention group includes a place to record daily updates on infant weight gain/loss, feeding, apneic and bradycardia episodes, and questions for the multidisciplinary healthcare team during bedside rounds. The intervention group Parent Journal also includes a checklist for the mother to record educational and support activities.

Immediately following infant discharge, and before the chart is dispatched to medical records, study nurses will collect data from the infant medical record and enter it into a web-based survey platform. In the event that the study nurses cannot complete infant discharge data collection (e.g., nursing staff transition), a research assistant will travel to the site and complete the infant discharge data collection.

When the infant(s) is/are age 2 months CA, the central coordinating site staff will collect and enter follow-up data from mothers, including information about infant development, feeding, hospital readmissions, and physician visits that are additional to the recommended health surveillance visits [52]. Data collectors cannot be blinded to group allocation at the 2-month CA follow-up because they require address and telephone number with area code to contact mothers. This information can reveal the site where the mother is currently living and the likely location of the birth of her preterm infant. Mothers have the option of completing the follow-up survey via their own computer using an on-line link, or via the telephone. For the duration of the trial, the central coordinating site staff will monitor events and activities that could be attributed to implementation of FICare or influence outcomes of the FICare study.

### Outcomes

*Primary outcome*: LOS in Level II NICUs, which will be measured as number of days between admission and discharge.

*Secondary outcomes*: (1) number of nosocomial infections between admission and discharge, (2) number of adverse events (e.g., medication and donor breast-milk errors) between admission and discharge, (3) number of times infants had feeding suspended (NPO) with concomitant interventions including initiation of intravenous starts and sepsis workups between admission and discharge, (4) number of hospital readmission rates, emergency room visits, and unplanned physician visits (additional to recommended pediatric health surveillance visits) [52] between discharge for birth hospitalization and 2 months CA, (5) scale scores on maternal psychosocial distress (stress, depression, anxiety) at baseline (enrollment), discharge and 2 months CA, (6) scale score on maternal confidence in caring for their infant at baseline, discharge, and 2 months CA, (7) proportion of infants receiving breast-milk feeding at discharge and 2 months CA; (8) scale scores on maternal breastfeeding self-efficacy at baseline, discharge, and 2 months CA, and (9) proportion of infants who score in the referral zone on a child developmental screener at 2 months CA. See Table 1 for scales and screener used in the admission, discharge, and 2-month CA follow-up.

**Table 1:** Study measures

Measure	Time point and metric	Description
Parental Stressor Scale: (PSS: NICU) [61]	Change in total and subscale scores between baseline and discharge	50-item scale that captures parental perceptions of stress in the NICU: (1) sights and sounds, (2) appearance and behavior of the infant, (3) impact on the parental role and relationship with the infant, and (4) parental relationship and communications with staff. Internal consistency (0.89 to 0.94 for the total scale) and test-retest (0.87) reliabilities are high
Parenting Stress Index - Short Form (4th ed.) [62]	Total and subscale scores at 2 months CA	36-item scale that captures general parenting stress as well as the three subscales of Parental Distress, Parent-Child Dysfunctional Interaction, and Difficult Child. Internal consistency (0.91 for Total Stress, 0.80 to 0.87 for subscales) and test-retest (0.84 for Total Stress, 0.68 to 0.84 for subscales) reliabilities are acceptable
Edinburgh Postnatal Depression Scale [63]	Change in total score and proportion in the clinical range (≥13) between baseline discharge and 2 months CA	The most commonly used pre- and postnatal depression screener validated for mothers. Consists of 10 items and has a sensitivity of 0.86 and specificity of 0.78, with a positive predictive value of 73%
State-Trait Anxiety Inventory (STAI) [64]^a^	Change in total score on State Anxiety between baseline, discharge and 2 months CA controlling for Trait Anxiety	40-item scale that captures dispositional/trait anxiety (20 items) and current state anxiety (20 items). Internal consistency (0.86 to 0.95) and test-retest (0.73 to 0.86) reliabilities are high. Scores on the STAI and PSS: NICU are correlated [61].
Perceived Maternal Parenting Self-Efficacy scale [65]^b^	Change in total score on between baseline discharge and 2 months CA	20-item measure of parenting self-efficacy validated for mothers of preterm infants. Captures maternal perceptions of ability to (1) give basic care, (2) elicit change in infant behavior, (3) recognize infant behavior, and (4) judge interactions with her infant. Exploratory factor analysis confirms four factors; internal consistency (0.91) and test-retest (0.96) reliabilities are high
Modified Breastfeeding Self-Efficacy Scale - Short Form [66]	Change in total score and subscale scores between baseline, discharge and 2 months CA	18-item scale validated for mothers of ill and/or preterm infants. Assesses a mother’s confidence in her ability to breastfeed when her infant is ready on four subscales: (1) performance accomplishments, (2) vicarious experience, (3) verbal persuasion, and (4) physiological responses. Internal consistency (0.88) is high
Ages and Stages Questionnaires (3rd ed.) (ASQ-3) [67]	Proportion of infants in the referral zone at 2 months CA	A widely used developmental screening instrument consisting of 21 age-appropriate questionnaires, with 30 items each, for use with infants and children 1 to 66 months of age. Assesses development in five domains: communication, gross motor, fine motor, problem-solving, and personal-social. For each domain, cut-off scores have been empirically derived for (a) appropriate development, (b) monitoring zone (≥1 and < 2 standard deviations below the mean), and (c) referral (2 standard deviations below the mean). The ASQ-3 has strong psychometric properties, including high sensitivity (0.86) and specificity (0.85) [68], and has been evaluated and recommended as an effective screener of global development in moderate and late preterm infants [23, 69, 70]

Note. ^a^At admission, both State and Trait forms are completed; at discharge and 2 months CA, only the State form is completed; ^b^Completed only by mothers who are breastfeeding

*Qualitative component*: (1) We will capture maternal experiences of FICare related to expectations of care, support for breastfeeding, and preparation for discharge, (2) we will capture HCP and hospital administrators’ experiences of FICare related to workload, staff satisfaction, and the various processes and structures at each site. These qualitative data will capture the process of implementation itself, and implementation quality will be coded within the five domains of the Consolidated Framework for Implementation Research [53]. The Damschroeder framework [53] will ensure a systematic and succinct assessment of implementation within a provincial healthcare organization.

*Economic evaluation*: the economic evaluation will compare direct and indirect costs between the FICare and standard care groups. Direct cost refers to health system costs: nursing time, clinical supplies, medications, hospitalizations, and physician visits additional to the recommended pediatric health surveillance visits in Canada [52]. The average hospital LOS for infants admitted to a Level II NICU in Alberta is 16 days, with an average per diem cost of $788 CAD [26]. In 2014, it cost Alberta approximately $43,459,776 CAD (3447 infants × $788 per day × 16 days) to provide Level II NICU care for moderate and late preterm infants. If FICare can reduce the hospital LOS by 10% (1.6 days), this would reduce costs to $39,113,798 CAD, which would be a cost saving of $4,345,978 CAD per year. This cost saving does not include hospital readmissions, emergency department, or physician visits additional to the recommended health surveillance visits (data for estimations not available at the time of grant submission). While direct healthcare system costs are of key importance to the health system, the indirect (out-of-pocket) costs of preterm birth have an impact on parents and other siblings in the family. For example, parents must pay out-of-pocket for parking, transportation, household support, and childcare for siblings while the infant is in hospital. In addition to time lost from work, parents may need to travel long distances to be near their hospitalized infant(s), and pay for food and accommodation. We will continue to capture direct healthcare system costs (e.g., hospital readmissions, emergency department visits) until the 2-month CA follow-up.

### Data collection, management, and analysis

#### Data collection

We define the primary outcome (LOS) as the time in days from birth (admission) to discharge. The attending physician determines infant discharge criteria based on infant wellness and parental caregiving capacity. We will measure secondary outcomes using a combination of health administrative data, parent-reported survey information, and self-reported scales. We will used semistructured interviews to explore mothers’ and HCP experiences of FICare. To estimate indirect costs and validate administrative data, we will ask parents to keep a daily diary of their expenses while their infant(s) is/are in the NICU.

Except in the rare case of maternal preference or equipment failure when mothers will complete a paper version of the questionnaires, mothers in both groups will use electronic tablets to complete questionnaires. FluidSurveys was planned as the data collection platform for this cRCT. When FluidSurveys was purchased by SurveyMonkey, which refused to extend research contracts, we needed to convert to Qualtrics (see https://www.qualtrics.com/). In the web-based survey, automatic skip patterns will ensure that mothers complete only relevant sections of questionnaires at each time point. For example, if a mother indicates that she does not intend to breastfeed, the section about breastfeeding self-efficacy is automatically bypassed. We gave careful consideration to the ethical principle of autonomy in requiring a response before a mother can proceed to the next question without skipping an answer. Except for scales with previously defined response categories, the majority of survey items include a “prefer not to answer” option.

#### Data management

We will collect data electronically. Once downloaded from the web-based platform and transferred to statistical software, we will examine data for missing values and patterns of missing values. Where appropriate and recommended by scale developers, we will replace missing values on scales or subscales with the mean score on that item for the group (intervention or control).

We will store data on University of Calgary servers that are backed-up daily. The servers are accessible only via university-managed, timed-out, password-protected computers. Permission for access to these computers can be granted only by investigator KB. These computers are located in dedicated, locked with key-entry research offices accessible only by research staff and designated graduate students. Investigator KB requires that all study investigators, research staff and students have (1) completed the Tri-Council Policy Statement: Ethical Conduct for Research Involving Humans (TCPS-2) certification, (2) a profile with their ethics board, and (3) signed a study-specific confidentiality agreement.

Once data are cleaned and the majority of analyses are complete, we will store anonymized data for those study participants who have consented. To facilitate data access by other qualified researchers, we consulted with PolicyWise Secondary Analysis to Generate Evidence (SAGE; formerly the Child Data Centre of Alberta) to ensure that consent and other data management processes are aligned with best practice for data storage (see https://policywise.com/initiatives/sage/).

#### Data analysis

We will aggregate outcomes (e.g., LOS, adverse events) across individuals, and compare the FICare group against the control group. We will perform intention-to-treat analyses. We will present numerical variables as means (*SD*) or medians (interquartile-range), and categorical variables as counts and percentages (with 95% confidence interval). We will use a significance level (*α*) of 0.05.

To compare LOS, the primary outcome, between FICare and standard care groups, we will use a *t*-test, taking into account clustering and the unequal cluster sizes. We will use a natural log transformation given that LOS has a skewed distribution [51]. For secondary analyses at the individual mother and infant level, we will perform a linear mixed-effects model to evaluate if the effect of FICare is confounded by demographic, infant and maternal health, and health system covariates. We will perform sensitivity analysis by removing mothers and/or their infants who were transferred between sites. We will analyze the secondary outcomes using *t*-tests for numerical outcomes and chi-square tests for categorical variables, adjusting for clustering. We will analyze secondary outcomes measured at more than one point in time using generalized linear-mixed models. We will not correct for multiple comparisons because we have only one primary research question. An additional secondary outcome available at the *cluster* level is adverse events including medication or human-milk administration errors captured through the AHS Reporting and Learning System. We will describe this outcome with ranges for each group.

#### Economic analyses

We will conduct the economic analyses using data collected prospectively alongside the study (i.e., during the hospital stay and at 2 months CA) from Parent Journals, study questionnaires, and administrative databases such as Data Integration, Measurement, and Research (DIMR) and discharge abstracts for hospitalizations, pharmacy, and laboratory services. First, we will conduct a cost analysis to estimate the total cost of FICare. We will identify the activities involved in the implementation of FICare and estimate the cost required to implement the intervention. Second, since the primary effectiveness outcome is LOS, which can be expressed in monetary terms (i.e., hospitalization cost), we will analyze the relative costs of FICare and standard care. The main outcome of the second objective will be the incremental difference in cost between FICare and standard care during hospitalization and at 2 months CA. We will conduct the analysis from two perspectives: the public healthcare payer and society. From the public payer’s perspective, we will include costs incurred to the Ministry, namely birth hospitalization, hospital readmissions, and emergency room and physician visits additional to recommended health surveillance visits for each infant enrolled in the study. In the detailed costs, we will include laboratory tests, imaging, and medications. From the societal perspective, we will include those costs incurred by families while their infant(s) is/are in the NICU, including parking, transportation, household support, missed work days, and childcare for siblings.

We will estimate total resource utilization cost as the product of resource use and unit cost. We will obtain unit costs from standard costing sources (e.g., Alberta Wage and Salary Survey) [54] and relevant organizations (e.g., the participating hospitals) [55]. After calculating total healthcare cost, we will analyze it as a dependent variable using regression to estimate the difference in expected healthcare cost between the two study groups. We will use regression to allow for the adjustment of potential confounders (e.g., demographic characteristics), and to calculate the clustered standard errors using the sandwich variance estimator attributed to Huber and White [56] in order to account for a clustered randomized clinical trial. We will calculate a 95% confidence interval to characterize the uncertainty. With a variety of different types of regression (e.g., ordinary least squares, generalized linear models), we will explore the impact of various modeling assumptions [57, 58]. In addition, we will compare parametric and non-parametric confidence intervals using bootstrapping [59]. In theory, an ordinary least-squares model produces unbiased estimates even if the data are skewed; however, different estimation methods (e.g., generalized linear models) and different uncertainty methods (e.g., non-parametric bootstrapping) will facilitate careful investigation of the impact various assumptions have on our conclusions. We will conduct a separate regression analysis for each perspective (public payer and society). In addition, we will use a net benefit regression framework [60] to compare the cost and effect of the FICare model against the standard of care with effect being the LOS after birth. This analysis would produce an incremental net benefit of the FICare model when compared to the standard of care at various willingness-to-pay values. Findings from the economic analysis of the FICare model may assist decision- and policy-makers in the delivery of health services to this important and specific population.

#### Data monitoring

We did not establish stopping guidelines or a data monitoring committee for this trial because FICare will be the standard of care at intervention sites, and is expected to enhance existing care, not cause harm. In addition, we did not power the study for interim analysis of the primary outcome.

Clinically, investigators have a professional responsibility to provide referrals for mothers who score at risk for postnatal depression (i.e., scored 13 or greater on the Edinburgh Postnatal Depression Scale (EPDS), or rated item #10 as 1, 2 or 3 points to indicate suicidal ideation). We check the database several times per week for EPDS scores in the clinical range. If a mother scores in the clinical range, the first author (KB) or a research associate with counseling psychology training will contact the study nurse if the infant is still in the NICU, or contact the mother at home if the infant(s) has/have been discharged. The contacts will include a brief explanation of the meaning of the EPDS score, and referral using the relevant path for the mother in hospital (typically, referred to social worker) or community (typically, referred to public health or family physician) for second-level screening, assessment and treatment, as appropriate. Similarly, for infants who screen at risk for developmental delay, we will contact mothers with a brief explanation and inquiry about how the infant is doing.

## Discussion

Our proposed pragmatic cRCT assesses an adapted version of FICare that will be implemented with moderate and late preterm infants admitted to Level II NICUs in Alberta. The results will inform policy decisions by providing evidence about the clinical effectiveness and costs of FICare. To accomplish knowledge translation (KT) goals, we will use both integrated and end-of-grant KT strategies. Integrated KT strategies require the inclusion of clinical and administrative leaders in the development and implementation of the intervention and the study. We will focus on strategies to embed components of FICare into existing structures and processes. For example, for the FICare nurses, we will identify aspects of the study that can be used to demonstrate learning to maintain annual nursing licensure. We will provide enhanced education sessions at intervention sites to ensure that changes in staff and competing priorities do not interfere with study implementation. We will use the Consolidated Framework for Implementation Research [53] to thoughtfully consider the barriers and facilitators that are unique to each intervention site. This work will enable our team to create a more fulsome user’s manual to support scale and broad implementation, should FICare prove to be effective. Our final integrated KT strategies focus on the inclusion of policy decision-makers in our large team meetings where we seek advice and guidance about the current initiatives in the healthcare system and alignment of our results with current directions. As the results from the study emerge, we will carefully observe for synergistic effects of FICare with other initiatives, as well as spin-offs such as proposals to adapt and implement FICare component in other populations and settings. Finally, our end-of-grant KT will include traditional strategies such as manuscripts and conference presentations. Requirements for authorship are outlined in our approved FICare Team Handbook, and follow the International Committee of Medical Journal Editors guidelines. Given the interest in the FICare study, and engagement with policy decision-makers at multiple levels in the healthcare system, we have already been invited to share the progress of our study with key stakeholder groups at provincial and national meetings.

## Trial status

On the date of manuscript submission, 352/659 mother/infant dyads were enrolled in the trial.

## Additional file

Additional file 1: SPIRIT Checklist. (DOCX 52 kb)

## Abbreviations

AHS: Alberta Health Services
CA: Corrected age
cRCT: Cluster randomized controlled trial
FICare: Family Integrated Care
GA: Gestational age
HCP: Healthcare providers
KT: Knowledge translation
LOS: Length of stay
NICU: Neonatal intensive care unit
