# Correction to: Family Integrated Care (FICare) in Level II Neonatal Intensive Care Units: study protocol for a cluster randomized controlled trial

**DOI:** 10.1186/s13063-020-04246-w

**Published:** 2020-03-19

**Authors:** Karen M. Benzies, Vibhuti Shah, Khalid Aziz, Wanrudee Isaranuwatchai, Luz Palacio-Derflingher, Jeanne Scotland, Jill Larocque, Kelly Mrklas, Esther Suter, Christopher Naugler, Henry T. Stelfox, Radha Chari, Abhay Lodha, Alberta FICare Level II NICU Study Team

**Affiliations:** 1 Faculty of Nursing, Department of Paediatrics, University of Calgary, PF 2278, 2500 University Drive, NW, Calgary, AB T2N 1N4 Canada; 2 Mount Sinai Hospital, Department of Paediatrics and Institute of Health Policy, Management and Evaluation, University of Toronto, Rm 19–231N, Mount, Sinai Hospital, 600 University Avenue, Toronto, ON M5G 1X5 Canada; 3 Edmonton Neonatal Program, Department of Pediatrics, Faculty of Medicine and Dentistry, University of Alberta, DTC 5027, Royal Alexandra Hospital, 10240 Kingsway NW, Edmonton, AB T5H 3V9 Canada; 4 Centre for Excellence in Economic Analysis Research (CLEAR), Li Ka Shing Knowledge Institute, St. Michael’s Hospital, Institute of Health Policy, Management and Evaluation, University of Toronto, 30 Bond Street, Toronto, ON M5B 1W8 Canada; 5 Department of Community Health Sciences, Faculty of Kinesiology, Sport Injury Prevention Research Center, University of Calgary, 2500 University Drive NW, PF2250G, Calgary, AB T2N 1N4 Canada; 6 Rockyview General Hospital, Unit 63, 7007 14th Street SW, Calgary, AB T2V 1P9 Canada; 7 DTC 5027, Royal Alexandra Hospital, 10240 Kingsway NW, Edmonton, AB T5H 3V9 Canada; 8 Knowledge for Change Unit, Research Innovation and Analytics, Alberta Health Services, 1103 South Tower, Foothills Medical Centre, 1403 29th Street NW, Calgary, AB T2N 2T9 Canada; 9 Faculty of Social Work, University of Calgary, Calgary, AB T2N 1N4 Canada; 10 Pathology and Laboratory Medicine, Faculty of Medicine, University of Calgary, Calgary, AB T2L 2K8 Canada; 11 General Pathology, Calgary Zone, Alberta Health Services; Calgary Laboratory Services, Alberta Health Services Laboratory Utilization Office, 9-3535 Research Road NW, Calgary, AB T2L 2K8 Canada; 12 Departments of Critical Care Medicine, Medicine and Community Health Sciences, Critical Care Strategic Clinical Network, University of Calgary, 3E18D, TRW Building, 3280 Hospital Drive NW, Calgary, AB T2N 4Z6 Canada; 13 Department of Obstetrics and Gynecology, Edmonton Zone, Alberta Health Services, University of Alberta, 5S131 -10240 Kingsway NW, Edmonton, AB T5H 3V9 Canada; 14 Department of Pediatrics and Community Health Sciences, Alberta Health Services, University of Calgary, C211-1403 29th Street NW, Calgary, AB T2N 2T9 Canada

**Correction to: Trials (2017) 18:467**

**https://doi.org/10.1186/s13063-017-2181-3**

After publication of our article [1], the authors have reported mathematical errors made in the sample size calculation for this cluster randomized controlled trial (cRCT) (Benzies et al. 2017). Independent statistician Peter Faris (PhD; Director, Research Facilitation, Analytics, Alberta Health Services) reviewed the sample size calculation for this cRCT and concluded that the statistician responsible for the original sample size calculation made a mathematical error when translating the absolute difference in length of stay (LOS) to a relative effect when estimating sample size using log-transformed LOS. As a consequence, the calculations were based on a 60% relative decrease in LOS rather than a 10% decrease. Additionally, we are unable to verify the intra-cluster correlation (ICC = 0.18) and standard deviation (SD = 0.235 in natural log scale) reported by the original statistician as the dataset used for these calculations is no longer available.

Retrospectively, using parameters from the cRCT (SD = 8, ICC = 0.085, mean LOS = 19 days, 10 sites, 65 mother participants per site), an absolute difference of 4.46 days in LOS (a 23.5% relative change in LOS) would have been required for the trial to achieve 80% power. Detailed calculations are available upon request from the first author. With only 10 available clusters in the province, a cRCT design was not feasible to achieve sufficient power to demonstrate a 10% difference between groups in LOS while adjusting for clustering. Moreover, the 10 clusters included all sites within the province, rather than a random selection of sites. Therefore, the analysis approach was modified to (1) assess the impact of the intervention within the province, and (2) account for site size, patient variation, and differences in mother and infant characteristics across the sites.
